# Association of angiotensin-converting enzyme gene I/D and CYP11B2 gene −344T/C polymorphisms with lone atrial fibrillation and its recurrence after catheter ablation

**DOI:** 10.3892/etm.2012.650

**Published:** 2012-07-31

**Authors:** XIAN-LING ZHANG, LI-QUN WU, XU LIU, YI-QING YANG, HONG-WEI TAN, XIN-HUA WANG, LI ZHOU, WEI-FENG JIANG, ZHENG LI

**Affiliations:** 1 Department of Cardiology, Shanghai Chest Hospital, Shanghai Jiaotong University School of Medicine;; 2 Department of Cardiology, Shanghai Tenth People’s Hospital, Tongji University;; 3 Department of Cardiology, Shanghai Ruijin Hospital;; 4 Department of Cardiovascular Research, Shanghai Chest Hospital, Shanghai Jiaotong University School of Medicine, Shanghai, P.R. China

**Keywords:** angiotensin-converting enzyme gene, CYP11B2, lone atrial fibrillation, recurrence, polymorphisms

## Abstract

The renin-angiotensin-aldosterone system (RAAS) plays a key role in atrial structural and electrical remodeling. The aim of this study was to investigate the potential associations of angiotensin-converting enzyme (ACE) gene insertion/ deletion (I/D) and aldosterone synthase (CYP11B2) gene −344T/C polymorphisms with the risk and recurrence of lone atrial fibrillation (AF). One hundred and ninety-three patients who underwent successful catheter ablation for lone AF were recruited. Two hundred and ninety-seven sinus rhythm subjects without a history of arrhythmia served as controls. The subjects were genotyped for ACE gene I/D and CYP11B2 gene −344T/C polymorphisms. Results showed that the ACE gene DD genotype and D allele were associated with a greater prevalence of lone AF (both P<0.01). In addition, the ACE gene DD genotype had a significantly larger left atrial dimension (LAD; 41.6±5.7 mm vs. 39.6±5.2 mm; P=0.043) and higher risk of AF recurrence [44.7% vs. 23.2%; odds ratio (OR), 2.68; 95% confidence interval (CI), 1.28–5.61; P=0.008] compared with the II+ID genotype in lone AF patients. After adjustment for a variety of risk factors, the ACE gene DD genotype had a 1.97-fold increased risk for lone AF (OR, 1.97; 95% CI, 1.15–3.37; P= 0.013) and 2.35-fold increased risk for AF recurrence (RR, 2.35; 95% CI, 1.10–5.04; P=0.028) compared with the ACE gene II+ID genotype. However, no correlation between the CYP11B2 gene −344T/C polymorphism and lone AF or its recurrence was observed in this cohort. In conclusion, the ACE gene DD genotype was associated with an increased incidence of lone AF and its recurrence following ablation, which was partly mediated by LAD.

## Introduction

Atrial fibrillation (AF) is one of the most common sustained cardiac arrhythmias in clinical practice, with a substantial morbidity and mortality rate (1). A growing body of evidence indicates that genetic factors are significant in the pathogenesis of lone AF and familial AF (2–4), with our previous studies indicating a genetic predisposition in familial AF (2,5). However, the mechanism underlying this disorder remains unclear.

The renin-angiotensin-aldosterone system (RAAS) blockers, not only reduce the risk of lone AF in normotensive and hypertensive patients, but also prevent AF recurrence (6–8). RAAS has been shown in several studies to promote inflammation, oxidation and fibrosis (9,10). In addition, experimental studies have shown that RAAS blockers prevent atrial structural and electrical remodeling (11,12), indicating a potential pathogenesis of lone AF. Previously, a 287-bp insertion/deletion (I/D) polymorphism of the angiotensin-converting enzyme (ACE) gene in intron 16 was identified. Both ACE plasma and tissue levels differ between patients with different ACE genotypes. The ACE gene DD genotype is linked to higher cellular ACE activity, leading to myocardial fibrosis (13,14). Mounting evidence suggests that ACE genetic predisposition may confer an increased risk of lone AF (3,15).

Aldosterone synthase (CYP11B2), a mitochondrial P450 oxidase mainly located in the zona glomerulosa of the adrenal cortex, is a key enzyme in aldosterone synthesis. One common polymorphism of the CYP11B2 gene (−344T/C) is located in the promoter region and appears to be functionally significant. The −344C allele has been shown to be associated with increased binding to steroidogenic transcription factor 1 (16) and has been linked to increased aldosterone synthase activity (17). Goette *et al* (18) demonstrated that aldosterone levels are elevated in patients with persistent AF, whereas restoration of sinus rhythm lowers serum aldosterone. In their study, Amir *et al* (19) reported that the CYP11B2 gene −344CC genotype was an independent predictor of AF in patients with heart failure.

Findings of recent study showed that polymorphisms on chromosome 4q25 modulate the risk for AF recurrence following catheter ablation (20). However, little is known about the potential genetic predisposition of the ACE gene I/D and CYP11B2 gene −344T/C polymorphisms with lone AF and its recurrence following catheter ablation. On the basis of this information and potentially the treatment of AF, we aimed to investigate the associations of the two polymorphisms with the risk of lone AF and its recurrence following catheter ablation in a Chinese Han population.

## Materials and methods

### Subjects

Between May 2007 and November 2009, 193 patients <65 years old, who underwent successful catheter ablation for symptomatic and lone drug-refractory AF, were recruited. Electrophysiological study and circumferential pulmonary vein ablation technique were described in detail in one of our previous studies (21). The lone AF (lone AF group, n=193) was diagnosed in patients who had AF on at least two occasions (>6 months apart) on a standard 12-lead electrocardiographic (ECG) recording and they all lacked known risk factors, including hypertension and structural heart disease. Patients were diagnosed with paroxysmal AF in 54%, persistent AF in 28% and longstanding persistent AF in 18% of cases. To evaluate the presence of structural heart disease, a detailed clinical history, physical examination, ECG, chest radiography, standard transthoracic echocardiography and transesophageal echocardiography were performed before the procedure to exclude left atrial thrombi, as we previously reported (22).

The control group consisted of 297 sinus rhythm subjects (control group, n=297) without history of arrhythmia, who underwent detailed physical screening examinations. Subjects with hypertension, diabetes mellitus, coronary artery disease, cardiomyopathy, valvular heart disease, left ventricular dysfunction [left ventricular ejection fraction (LVEF) <50%], thyroid diseases, renal failure requiring dialysis and serious life-threatening illnesses or inflammation in the last 6 months were excluded from the study. None of the recruited subjects were given class I or III antiarrhythmic drugs prior to enrollment. The two groups had no history of familial arrhythmias. The study protocol was reviewed andapproved be the Shanghai Chest Hospital Ethics Committee and written informed consent was obtained from all participants prior to recruitment.

Blood samples were collected after overnight fasting and stored at −80°C. Serum levels of fasting glucose, total cholesterol, triglycerides, blood urea nitrogen, creatinine and uric acid were measured (Hitachi 912 analyser; Roche Diagnostics, Mannheim, Germany).

Transthoracic echocardiography examinations were performed in all subjects with a 2.5-MHz transducer attached to a Doppler echocardiography machine. Left ventricular end-systolic diameter (LVESD), left ventricular end-diastolic diameter (LVEDD) and left atrial dimension (LAD), as well as septal wall thickness (SWT) and posterior wall thickness (PWT) at end-diastole were measured in the parasternal long axis view, using two-dimensional guided M-mode echocardiography according to the recommendations for chamber quantification (23). The LVEF was determined from the parasternal long axis view using the Teichholz method (23).

### Genotyping

Genomic DNA was extracted from the peripheral blood leukocytes of all subjects using standard protocols with the Wizard^®^ genomic DNA purification kit (Promega, Madison, WI, USA). Subjects were genotyped for the ACE gene I/D polymorphism using polymerase chain reaction (PCR). The primer sequences used were: forward 5′-CTGGAGACCACTCCCATCCTTTCT-3′ and reverse 5′-GATGTGGCCATCACATTCGTCAGAT-3′. The PCR products were resolved by electrophoresis in a 1.5% agarose gel. CYP11B2 gene −344T/C polymorphisms (rs1799998) were genotyped by PCR amplification and restriction fragment length polymorphism analysis. Primer sequences (19) used were: forward 5′-CAGGAGGAGACCCCATGTGA-3′ and reverse 5′-CCTCCACCCTGTTCAGCCC-3′, followed by digestion with *Hae*III (New England BioLabs, Inc., Ipswich, MA, USA). The digestion products were resolved by electrophoresis in a 2% agarose gel. For all polymorphisms, the genotype was confirmed by two independent technicians and any discrepancies were confirmed by repeat genotyping.

### Follow-up

As we have previously reported (22), the patients were prospectively followed-up for >3 months (blanking period) and routinely received low molecular weight heparin injections for 3–5 days and warfarin anticoagulation for at least 1 month, as well as proton pump inhibitors for 4 weeks. Subsequent to pulmonary vein isolation, the patients were administered class III antiarrhythmic drugs with amiodarone 200–400 mg/day or sotalol 80–160 mg/day for 1–3 months. The severity of symptoms was evaluated monthly by telephone and patients were asked to record their ECG when they had any symptoms indicating the onset of AF. A 48-h Holter recording was performed 3 months after the procedure to document any form of atrial arrhythmia. An AF recurrence was defined as a documented atrial arrhythmia episode lasting >30 sec at 3 months after ablation (20).

### Statistical analysis

Continuous variables were expressed as the mean ± SD and categorical variables were presented as frequencies. Normal distribution was evaluated with the Kolmogorov-Smirnov test. A logarithmic transformation was performed on the continuous variables of non-normal distribution prior to statistical calculations to achieve normal distribution. The comparisons between groups were performed using unpaired Student’s t-tests, differences in continuous variables across genotype groups were tested using the analysis of variance (ANOVA). Genotype and allele frequencies were compared among study groups using the Chi-square test. The Hardy-Weinberg equilibrium for each polymorphism was also assessed using the Chi-square test. For the genotypes present in significantly different frequencies, a multivariate logistic regression model was performed to estimate the odds ratio (OR) and the corresponding 95% confidence interval (CI), adjusting for the covariates. P≤0.05 was considered to indicate a statistically significant result. All analyses were performed with SPSS for Windows 13.0 (SPSS, Inc., Chicago, IL, USA).

## Results

### Patient characteristics and AF recurrence

Table I lists the baseline clinical characteristics and biochemical measurements in the lone AF and control groups, respectively. Between the two groups age was matched (P=0.20) and there was no significant difference in terms of smoking status, systolic and diastolic blood pressure, fasting glucose, total cholesterol, triglycerides or blood urea nitrogen between the two groups. However, there were more males and alcohol drinkers in the lone AF group (P=0.026 and P=0.044, respectively) than in the control group. We also observed that the AF cases were more likely to have poorer renal function indicated by greater uric acid, creatinine and higher body mass index (BMI) compared with the controls, although the latter two variables did not reach statistical significance (creatinine and BMI, P=0.09 and P=0.06, respectively). As expected, lone AF patients had significantly larger LAD, LVEDD, and lower LVEF than the control subjects (all P<0.05). However, we identified no significant difference with regard to the LVESD, SWT or PWT in this cohort (Table I).

Based on the ECG/Holter documentation, of the 193 patients with successful pulmonary vein isolation, 53 patients developed AF recurrence (recurrence group) 3 months after the procedure, while 140 cases (non-recurrence group) retained sinus rhythm. We observed that the recurrence group patients were older, had a higher BMI, larger LAD and lower LVEF compared with those in the non-recurrence group (all P<0.05; Table I). No significant difference was found between the two groups with respect to other risk factors (Table I).

### Association of the two polymorphisms with AF and its recurrence

The distribution of all genotypes was in Hardy-Weinberg equilibrium. Genotype frequencies for the ACE gene I/D polymorphism are reported in Table II. We observed that the ACE gene DD genotype had a 2.27-fold increased risk for lone AF compared with the ACE gene II+ID genotype (OR, 2.27; 95% CI, 1.34–3.82; P= 0.002). The frequencies of the ACE gene D allele in the lone AF and control groups were 42.2 and 33.2%, respectively. Similarly, the ACE gene D allele had a significantly higher incidence in lone AF patients than in the control subjects (42.2% vs. 33.2%; OR, 1.47; 95% CI, 1.13–1.92; P=0.004). With regard to the CYP11B2 gene −344T/C polymorphism, −344TT, −344TC and −344CC genotypes were distributed according to the following percentages: lone AF group 50.3, 39.9, 9.8% and control groups 46.8, 41.1, 12.1%, respectively. The minor C allele frequency of the CYP11B2 gene −344T/C polymorphism in the lone AF and control groups was 29.8 and 32.7%, respectively. By contrast, we did not find statistically significant differences in the genotype and allele distribution of −344T/C polymorphism between lone AF patients and controls (Table II).

**Figure 1:**
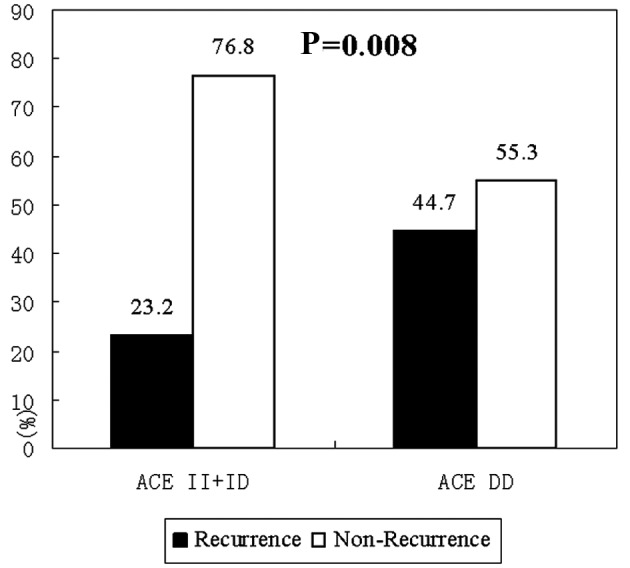
Recurrence and non-recurrence of AF following catheter ablation in patients with lone AF by ACE gene DD and II+ID genotype. ACE, angiotensin-converting enzyme; AF, atrial fibrillation.

We further examined the association between the ACE gene I/D polymorphism and baseline parameters, as well as AF recurrence. As a result, the ACE gene DD genotype had significantly larger LAD compared with the II+ID genotype (39.6±5.2 mm vs. 41.6±5.7 mm, P=0.043; Table III). In addition, we observed that the ACE gene DD genotype had a higher risk of AF recurrence compared with the II+ID genotype (23.2% vs. 44.7%; RR, 2.68; 95% CI, 1.28–5.61; P=0.008; Table III and Fig. 1). However, with regard to the −344T/C polymorphism of CYP11B2 gene, no significant difference was observed between this polymorphism and the baseline parameters (Table IV).

### Regression analysis

In order to predict determinants of lone AF, we included relevant clinical characteristics in a multivariate logistic regression model. After adjustment for gender, age, smoking status, alcohol intake, BMI, systolic and diastolic blood pressure, creatinine, uric acid, LVEDD, LAD and LVEF, we observed that male gender, LAD and ACE gene DD genotype were the independent risk factors for lone AF, and that the ACE gene DD genotype had a 1.97-fold increased risk for lone AF compared with the ACE gene II+ID genotype (OR, 1.97; 95% CI, 1.15–3.37; P= 0.013; Table V). To investigate AF recurrence, we performed a multivariate stepwise logistic regression model, including variables such as age, gender, BMI, LVEF and LAD, and observed that the ACE gene DD genotype had a 2.35-fold increased risk for AF recurrence (RR, 2.35; 95% CI, 1.10–5.04; P= 0.028) compared with the ACE gene II+ID genotype. Among the other variables, only LAD had a significant effect on the risk of AF recurrence.

## Discussion

In the present study, we investigated the associations of the ACE gene I/D and CYP11B2 gene −344T/C polymorphisms with lone AF and its recurrence following catheter ablation. To the best of our knowledge, no similar study has been published that explores the associations between the two polymorphisms and AF recurrence after the procedure. Based on the analysis of 193 lone AF patients and 297 sinus rhythm subjects, we observed that the ACE gene DD genotype and D allele were associated with a greater prevalence of lone AF, which was consistent with previous studies (3,24). This association was independent of a variety of risk factors, which might be potential confounders.

It is known that AF is associated with a significant risk of recurrence following catheter ablation, even if serial antiarrhythmic drug treatment is administered (25,26). Previous studies have shown that RAAS blockers are effective in preventing AF recurrence in both normotensive (6) and hypertensive patients (27,28). Therefore, we prospectively followed-up the lone AF patients for more than 3 months. Additionally, the correlation between the two polymorphisms and lone AF recurrence was examined. In our cohort, the univariate analysis revealed that the ACE gene DD genotype had a 2.68-fold increased risk of AF recurrence compared with the II+ID genotype. This significance remained (RR, 2.35; 95% CI, 1.10–5.04; P=0.028) after controlling for the covariates. In addition, LAD was another independent predictor of AF recurrence, which was consistent with previous reports (29,30). Therefore, we suggest that the ACE gene I/D polymorphism is potentially a susceptibility locus for the risk of lone AF and its recurrence in this Chinese cohort.

It is noteworthy that the ACE gene DD genotype had statistically increased LAD compared with the II+ID genotype, suggesting that the ACE gene D allele is likely responsible for LAD in lone AF patients. LAD was associated with a significantly increased risk for lone AF in this cohort. Consequently, the results of our study suggest that the association of the ACE gene I/D polymorphism with lone AF and its recurrence is likely to be mediated, at least in part, by the effects of the ACE gene I/D polymorphism on LAD.

However, the exact molecular mechanism underlying this disorder remains unknown and available scientific evidence has not elucidated gene-gene and gene-environmental interactions. One potential explanation is that the ACE gene I/D polymorphism is a functional variant located in intron 16 of the ACE gene, both ACE plasma and tissue levels differ in subjects with different ACE genotypes, and ACE DD genotype is linked to higher cellular ACE activity leading to myocardial fibrosis (13,14). Angiotensin II, which is the main product and effector of ACE, has proinflammatory actions and leads to the production of reactive oxygen species and a series of inflammatory cytokines (31). In addition, it activates fibroblasts leading to fibrosis and scar formation, which is crucial in the electrical and structural remodeling that occurs in the left atrium during AF (9). We also cannot exclude the possibility of an unidentified susceptibility gene polymorphism in linkage disequilibrium with ACE gene affects the expression of it, and in turn, promotes the occurrence and recurrence of AF.

Previous studies had reported the inconsistent associations between CYP11B2 gene −344T/C polymorphism and AF. Amir *et al* (19) reported that the CYP11B2 gene −344CC genotype was an independent predictor of AF in patients with heart failure. However, a recent study by Huang *et al* (32) reported that no correlation between the CYP11B2 gene −344T/C polymorphism and AF was identified in a Chinese Han population with hypertensive heart disease. In our cohort, no association of CYP11B2 gene −344T/C polymorphism with lone AF or relapses was found, consistent with the study by Huang *et al* (32). The reasons for these discrepancies are unclear but may have been caused by factors including allelic heterogeneity, ethnic discrepancy and criteria for recruitment, including gender, age and heart disease. Previous studies have shown significant differences in the allele frequency of the CYP11B2 gene −344T/C polymorphism between various ethnic or geographical origins (33). It is noteworthy that our cases were all lone AF patients of Chinese descent who lacked structural heart disease, which was not in accordance with the study by Amir *et al* (19).

Our study had certain limitations. First, the sample size of the AF recurrence group was relatively small and the observation period was relatively short. Second, we had no ACE plasma data in this study, however, ACE gene I/D polymorphism is a functional variant, and experimental studies have shown that the presence of the D allele is associated with higher levels of plasma ACE (14). Third, even ‘control’ individuals may have AF, a fact that may have been compounded by the recruitment of controls from atypical symptoms or electrocardiogram.

In summary, the present study provides evidence that the ACE gene DD genotype affects susceptibility to lone AF and its recurrence subsequent to ablation among the Chinese Han population. This association was independent of established risk factors, but appears to be mediated, at least in part, by effects of the ACE gene DD genotype on LAD. This novel finding expands the understanding of molecular mechanisms underlying lone AF and this genetic factor, as well as potentially the treatment of ACE blockers on AF and its recurrence. However, long-term follow-up studies with larger sample sizes are required to assess the significance of RAAS-related gene polymorphisms.

## Figures and Tables

**Table I:** Baseline clinical characteristics and biochemical measurements.

Variables	Control (n=297)	Lone AF (n=193)	P-value	Non-recurrence (n=140)	Recurrence (n=53)	P-value
Males, n (%)	148 (49.8)	116 (60.1)	0.026	82 (58.6)	34 (64.2)	0.48
Age (years)	56.3±6.2	57.1±7.3	0.20	56.4±7.5	58.8±6.3	0.037
Smoking, n (%)	52 (17.5)	43 (22.3)	0.19	28 (20.0)	15 (28.3)	0.22
Alcohol, n (%)	41 (13.8)	40 (20.7)	0.044	25 (17.9)	15 (28.3)	0.11
Body mass index (kg/m^2^)	24.7±2.5	25.1±2.9	0.06	24.9±2.9	25.8±2.7	0.039
Systolic blood pressure (mmHg)	120.5±11.4	121.5±11.4	0.34	120.9±11.9	122.9±10.2	0.27
Diastolic blood pressure (mmHg)	74.7±7.7	75.0±8.5	0.68	75.2±8.5	74.4±8.3	0.53
Fasting glucose (mmol/l)	5.12±0.57	5.17±0.57	0.29	5.14±0.57	5.26±0.57	0.17
Total cholesterol (mmol/l)	4.39±0.76	4.40±0.77	0.99	4.35±0.76	4.52±0.78	0.16
Triglycerides (mmol/l)	1.55±0.52	1.48±0.50	0.10	1.48±0.50	1.47±0.51	0.90
Blood urea nitrogen (mmol/l)	5.40±1.33	5.57±1.32	0.15	5.54±1.30	5.68±1.37	0.50
Creatinine (*μ*mol/l)	73.5±15.7	76.0±16.8	0.09	75.8±16.8	76.5±16.8	0.80
Uric acid (*μ*mol/l)	315±67	331±72	0.012	328±68	341±82	0.25
LV end-systolic diameter (mm)	29.2±3.2	29.6±4.6	0.32	29.8±5.0	29.3±3.5	0.50
LV end-diastolic diameter (mm)	47.2±3.9	48.1±4.2	0.022	47.8±4.2	48.8±4.1	0.13
Left atrial dimension (mm)	38.6±3.2	40.0±5.3	<0.001	39.3±4.6	41.9±6.6	0.002
Septal wall thickness (mm)	9.0±1.0	9.1±1.1	0.85	9.0±1.2	9.1±1.1	0.55
Posterior wall thickness (mm)	8.7±1.0	8.7±1.0	0.42	8.7±1.0	8.9±1.1	0.24
LV ejection fraction (%)	63.6±6.5	62.5±5.1	0.040	62.9±5.1	61.3±4.8	0.040

Values are presented as the mean ± SD or n (%). AF, atrial fibrillation; LV, left ventricular.

**Table II:** Genotype and allele frequencies of ACE gene I/D and CYP11B2 gene −344T/C polymorphisms.

	ACE gene I/D polymorphism					CYP11B2 gene −344T/C polymorphism			
	II	ID	DD	II+ID	D	TT	TC	CC	C
Control n (%)	129 (43.4)	139 (46.8)	29 (9.8)	268 (90.2)	197 (33.2)	139 (46.8)	122 (41.1)	36 (12.1)	194 (32.7)
Lone AF n (%)	68 (35.2)	87 (45.1)	38 (19.7)	155 (80.3)	163 (42.2)	97 (50.3)	77 (39.9)	19 (9.8)	115 (29.8)
P-value		0.005		0.002^a^	0.004		0.647		0.345

a P<0.01 vs. DD genotype. AF, atrial fibrillation; ACE, angiotensin-converting enzyme; I/D, insertion/deletion.

**Table III:** Patient characteristics with respect to ACE gene I/D genotype.

Variables	II+ID (n=155)	DD (n=38)	P-value
Males, n (%)	92 (59.4)	24 (63.2)	0.67
Age (years)	57.0±7.3	57.6±7.3	0.66
Body mass index (kg/m^2^)	25.0±2.8	25.4±3.0	0.45
Systolic blood pressure (mmHg)	120.8±11.3	124.4±11.7	0.08
Diastolic blood pressure (mmHg)	74.8±8.8	75.8±7.1	0.51
Fasting glucose (mmol/l)	5.15±0.59	5.25±0.50	0.34
Total cholesterol (mmol/l)	4.39±0.78	4.43±0.73	0.75
Triglycerides (mmol/l)	1.48±0.51	1.48±0.48	0.95
Blood urea nitrogen (mmol/l)	5.54±1.31	5.71± 1.38	0.50
Creatinine (*μ*mol/l)	75.9±17.1	76.4±15.6	0.88
Uric acid (*μ*mol/l)	331±72	335±72	0.73
Recurrence, n (%)	36 (23.2)	17 (44.7)	0.008
LV end-systolic diameter (mm)	29.6±4.8	29.7±3.8	0.92
LV end-diastolic diameter (mm)	48.0±4.2	48.2±4.2	0.79
Left atrial dimension (mm)	39.6±5.2	41.6±5.7	0.043
Septal wall thickness (mm)	9.0±1.1	9.1±1.0	0.66
Posterior wall thickness (mm)	8.7±1.1	8.8±0.9	0.56
LV ejection fraction (%)	62.8±4.9	61.1±5.5	0.052

LV, left ventricular; ACE, angiotensin-converting enzyme.

**Table IV:** Patient characteristics with respect to CYP11B2 gene −344T/C genotype.

Variables	TT (n=97)	TC (n=77)	CC (n=19)	P-value
Male, n (%)	59 (60.8)	46 (59.7)	11 (57.9)	0.97
Age (years)	57.0±7.0	57.2±7.6	56.9±7.9	0.99
Body mass index (kg/m^2^)	25.2±2.8	24.8±2.8	25.8±3.4	0.37
Systolic blood pressure (mmHg)	122.1±11.0	121.8±11.8	116.7±11.7	0.16
Diastolic blood pressure (mmHg)	75.6±8.6	75.1±8.3	71.3±7.9	0.12
Fasting glucose (mmol/l)	5.14±0.57	5.22±0.58	5.14±0.57	0.65
Total cholesterol (mmol/l)	4.37±0.75	4.48±0.77	4.20±0.84	0.35
Triglycerides (mmol/l)	1.44±0.51	1.52±0.48	1.49±0.57	0.62
Blood urea nitrogen (mmol/l)	5.59±1.28	5.58±1.40	5.46±1.19	0.93
Creatinine (*μ*mol/l)	75.3±17.0	77.4±17.5	74.0±12.2	0.62
Uric acid (*μ*mol/l)	330±72	334±74	328±69	0.91
Recurrence, n (%)	28 (28.9)	20 (26.0)	5 (26.3)	0.91
LV end-systolic diameter (mm)	29.8±4.1	29.3±5.0	29.7±5.7	0.76
LV end-diastolic diameter (mm)	48.4±4.3	47.7±4.0	47.6±4.2	0.42
Left atrial dimension (mm)	40.4±5.1	39.6±5.4	39.7±6.6	0.64
Septal wall thickness (mm)	9.2±1.2	8.9±1.1	8.8±1.0	0.17
Posterior wall thickness (mm)	8.8±1.1	8.7±1.0	8.5±0.8	0.33
LV ejection fraction (%)	62.5±5.2	62.5±4.8	62.1±5.8	0.94

LV, left ventricular.

**Table V:** Multivariate logistic regression model for lone atrial fibrillation.

Variable	OR	95% CI	P-value
Male	1.47	1.01–2.13	0.045
Left atrial dimension	1.08	1.03–1.13	0.002
ACE gene DD genotype	1.97	1.15–3.37	0.013

OR, odds ratio; 95% CI, 95% confidence interval; ACE, angiotensin-converting enzyme.

## References

[1] Benjamin EJ, Levy D, Vaziri SM, D’Agostino RB, Belanger AJ, Wolf PA (1994). Independent risk factors for atrial fibrillation in a population-based cohort. The Framingham Heart Study. JAMA.

[2] Jiang J, Shen F, Fang W, Liu X, Yang YQ (2011). Novel GATA4 mutations in lone atrial fibrillation. Int J Mol Med.

[3] Fatini C, Sticchi E, Gensini F, Gori AM, Marcucci R, Lenti M, Michelucci A, Genuardi M, Abbate R, Gensini GF (2007). Lone and secondary nonvalvular atrial fibrillation: role of a genetic susceptibility. Int J Cardiol.

[4] Yang YQ, Liu X, Zhang XL, Wang XH, Tan HW, Shi HF, Jiang WF, Fang WY (2010). Novel connexin40 missense mutations in patients with familial atrial fibrillation. Europace.

[5] Yang Y, Xia M, Jin Q, Bendahhou S, Shi J, Chen Y, Liang B, Lin J, Liu Y, Liu B (2004). Identification of a KCNE2 gain-of-function mutation in patients with familial atrial fibrillation. Am J Hum Genet.

[6] Belluzzi F, Sernesi L, Preti P, Salinaro F, Fonte ML, Perlini S (2009). Prevention of recurrent lone atrial fibrillation by the angiotensin-II converting enzyme inhibitor ramipril in normotensive patients. J Am Coll Cardiol.

[7] Tayebjee MH, Creta A, Moder S, Hunter RJ, Earley MJ, Dhinoja MB, Schilling RJ (2010). Impact of angiotensin-converting enzyme-inhibitors and angiotensin receptor blockers on long-term outcome of catheter ablation for atrial fibrillation. Europace.

[8] Klemm HU, Heitzer T, Ruprecht U, Meinertz T, Ventura R (2010). Impact of angiotensin-converting enzyme inhibitors and angiotensin II receptor blockers on the long-term outcome after pulmonary vein isolation for paroxysmal atrial fibrillation. Cardiology.

[9] Marchesi C, Paradis P, Schiffrin EL (2008). Role of the reninangiotensin system in vascular inflammation. Trends Pharmacol Sci.

[10] Rahman ST, Lauten WB, Khan QA, Navalkar S, Parthasarathy S, Khan BV (2002). Effects of eprosartan versus hydrochlorothiazide on markers of vascular oxidation and inflammation and blood pressure (renin-angiotensin system antagonists, oxidation, and inflammation). Am J Cardiol.

[11] Kumagai K, Nakashima H, Urata H, Gondo N, Arakawa K, Saku K (2003). Effects of angiotensin II type 1 receptor antagonist on electrical and structural remodeling in atrial fibrillation. J Am Coll Cardiol.

[12] Nakashima H, Kumagai K, Urata H, Gondo N, Ideishi M, Arakawa K (2000). Angiotensin II antagonist prevents electrical remodeling in atrial fibrillation. Circulation.

[13] Danser AH, Schalekamp MA, Bax WA, van den Brink AM, Saxena PR, Riegger GA, Schunkert H (1995). Angiotensin-converting enzyme in the human heart. Effect of the deletion/insertion polymorphism. Circulation.

[14] Rigat B, Hubert C, Alhenc-Gelas F, Cambien F, Corvol P, Soubrier F (1990). An insertion/deletion polymorphism in the angiotensin I-converting enzyme gene accounting for half the variance of serum enzyme levels. J Clin Invest.

[15] Watanabe H, Kaiser DW, Makino S, MacRae CA, Ellinor PT, Wasserman BS, Kannankeril PJ, Donahue BS, Roden DM, Darbar D (2009). ACE I/D polymorphism associated with abnormal atrial and atrioventricular conduction in lone atrial fibrillation and structural heart disease: implications for electrical remodeling. Heart Rhythm.

[16] White PC, Slutsker L (1995). Haplotype analysis of CYP11B2. Endocr Res.

[17] Brand E, Chatelain N, Mulatero P, Fery I, Curnow K, Jeunemaitre X, Corvol P, Pascoe L, Soubrier F (1998). Structural analysis and evaluation of the aldosterone synthase gene in hypertension. Hypertension.

[18] Goette A, Hoffmanns P, Enayati W, Meltendorf U, Geller JC, Klein HU (2001). Effect of successful electrical cardioversion on serum aldosterone in patients with persistent atrial fibrillation. Am J Cardiol.

[19] Amir O, Amir RE, Paz H, Mor R, Sagiv M, Lewis BS (2008). Aldosterone synthase gene polymorphism as a determinant of atrial fibrillation in patients with heart failure. Am J Cardiol.

[20] Husser D, Adams V, Piorkowski C, Hindricks G, Bollmann A (2010). Chromosome 4q25 variants and atrial fibrillation recurrence after catheter ablation. J Am Coll Cardiol.

[21] Wang X, Liu X, Shi H, Tan H, Jiang W, Wang Y, Yang G, Zhou L (2011). Early recurrences after paroxysmal atrial fibrillation ablation: when is the proper timing for reablation?. Pacing Clin Electrophysiol.

[22] Liu X, Tan H, Wang X, Shi H, Li Y, Li F, Zhou L, Gu J (2010). Efficacy of catheter ablation and surgical CryoMaze procedure in patients with long-lasting persistent atrial fibrillation and rheumatic heart disease: a randomized trial. Eur Heart J.

[23] Lang RM, Bierig M, Devereux RB, Flachskampf FA, Foster E, Pellikka PA, Picard MH, Roman MJ, Seward J, Shanewise J (2006). Recommendations for chamber quantification. Eur J Echocardiogr.

[24] Ravn LS, Benn M, Nordestgaard BG, Sethi AA, Agerholm-Larsen B, Jensen GB, Tybjaerg-Hansen A (2008). Angiotensinogen and ACE gene polymorphisms and risk of atrial fibrillation in the general population. Pharmacogenet Genomics.

[25] Roux JF, Zado E, Callans DJ, Garcia F, Lin D, Marchlinski FE, Bala R, Dixit S, Riley M, Russo AM (2009). Antiarrhythmics after ablation of atrial fibrillation (5A study). Circulation.

[26] Balk EM, Garlitski AC, Alsheikh-Ali AA, Terasawa T, Chung M, Ip S (2010). Predictors of atrial fibrillation recurrence after radio-frequency catheter ablation: a systematic review. J Cardiovasc Electrophysiol.

[27] Fogari R, Derosa G, Ferrari I, Corradi L, Zoppi A, Lazzari P, Santoro T, Preti P, Mugellini A (2008). Effect of valsartan and ramipril on atrial fibrillation recurrence and P-wave dispersion in hypertensive patients with recurrent symptomatic lone atrial fibrillation. Am J Hypertens.

[28] Staszewsky L, Wong M, Masson S, Raimondi E, Gramenzi S, Proietti G, Bicego D, Emanuelli C, Pulitanò G, Taddei F (2011). Left atrial remodeling and response to valsartan in the prevention of recurrent atrial fibrillation: the GISSI-AF echo-cardiographic substudy. Circ Cardiovasc Imaging.

[29] Shin SH, Park MY, Oh WJ, Hong SJ, Pak HN, Song WH, Lim DS, Kim YH, Shim WJ (2008). Left atrial volume is a predictor of atrial fibrillation recurrence after catheter ablation. J Am Soc Echocardiogr.

[30] Arriagada G, Berruezo A, Mont L, Tamborero D, Molina I, Coll-Vinent B, Vidal B, Sitges M, Berne P, Brugada J, GIRAFA (Grup Integrat de Recerca en Fibril.lació Auricular) Investigators (2008). Predictors of arrhythmia recurrence in patients with lone atrial fibrillation. Europace.

[31] Brasier AR, Recinos A, Eledrisi MS (2002). Vascular inflammation and the renin-angiotensin system. Arterioscler Thromb Vasc Biol.

[32] Huang M, Gai X, Yang X, Hou J, Lan X, Zheng W, Chen F, He J (2009). Functional polymorphisms in ACE and CYP11B2 genes and atrial fibrillation in patients with hypertensive heart disease. Clin Chem Lab Med.

[33] Barbato A, Russo P, Siani A, Folkerd EJ, Miller MA, Venezia A, Grimaldi C, Strazzullo P, Cappuccio FP (2004). Aldosterone synthase gene (CYP11B2) C−344T polymorphism, plasma aldosterone, renin activity and blood pressure in a multi-ethnic population. J Hypertens.

